# Qualitative Evaluation: GRECC Connect as a Method of Delivering Health Care to Rural Older Veterans

**DOI:** 10.1093/geroni/igab046.454

**Published:** 2021-12-17

**Authors:** Lauren Moo, William Hung, Eileen Dryden, Camilla Pimentel, Laura Kernan, Kathryn Nearing

**Affiliations:** 1 VA Bedford Health Care System, Bedford, Massachusetts, United States; 2 Veterans Health Administration, New York City, New York, United States; 3 Veterans Health Administration, Bedford, Massachusetts, United States; 4 VA Bedford Healthcare System, Bedford, Massachusetts, United States; 5 Veterans Health Administration, Denver, Colorado, United States

## Abstract

The VA Office of Rural Health-funded GRECC Connect program uses telehealth modalities to provide geriatric specialty care to rural older veterans and education to clinicians in VA Community-based outpatient clinics (CBOCs). Qualitative evaluation of GRECC Connect has included interviews with three stakeholder groups: geriatrics specialty teams at 15 hub medical centers, rural CBOC staff, and patients/family caregivers. CBOC staff interviews included 50 individuals from 13 different CBOCs. Staff roles included clinic managers, social workers, psychologists, physicians, nurses, and telehealth technicians. Older veterans who had recently been involved in a GRECC Connect video visit were also invited to share their views on the visit. By including multiple perspectives on the program, we are better positioned to increase reach, access, and improve care for older rural veterans.

